# The Outcome of COVID-19 Infection in Patients With Gastrointestinal Diseases: An Experience at a Tertiary Center

**DOI:** 10.7759/cureus.35629

**Published:** 2023-03-01

**Authors:** Irgen Tafaj, Liri Cuko, Qazim Çili, Arlinda Hysenj, Edite Sadiku

**Affiliations:** 1 Gastroenterology and Hepatology, Mother Teresa University Hospital Center, Tirana, ALB

**Keywords:** albania, systemic inflammation indices, liver cirrhosis, digestive disease, covid-19

## Abstract

Objective: Observing the impact of the coronavirus disease 2019 (COVID-19) pandemic on digestive diseases in hospitalized patients at the Department of Gastroenterology-Hepatology in “Mother Teresa” University Hospital Center (UHC),Tirana.

Methods: This retrospective study was carried out from June 2020 to December 2021 involving 41 cases of patients >18 years who were positive for COVID-19 infection detected by RT-PCR (Reverse Transcription-Polymerase Chain Reaction) assays of nasopharyngeal swab specimens. The severity of COVID-19 infection was evaluated by hematological/biochemical parameters, blood oxygenation/need for oxygen, radiological data on pulmonary CT imaging.

Results: Out of 2527 hospitalized cases, 1.6% (41) were positive for the infection. The average age was 60.05 +/- 15.008 years. The group of age with more patients (48.8%) was 41-60 years. Infected males were higher than females (p<0.001). Out of the total, 21% were vaccinated at the diagnosis. Most patients came from urban areas, more than a half from the capital. Frequency of the digestive diseases was: cirrhosis 31.7%, pancreatitis 21.9%, alcoholic liver disease 21.9%, gastrointestinal hemorrhage 19.5%, digestive cancer 14.6%, biliary diseases 7.3%, inflammatory bowel disease (IBD) 2.4%, other digestive diseases 4.8%. Fever (90%) and fatigue (78.04%) were the dominant clinical signs.* *

Biochemical and hematological parameters showed elevation of average value of aspartate amino transferase (AST), alanine transaminase (ALT) (AST>ALT, p<0.001), and bilirubin in all the patients. Higher levels of creatinine and significantly predictive value of systemic inflammation indices NLR (neutrophil to lymphocyte ratio ) and MLR (monocyte to lymphocyte ratio) were found in the fatality cases.

Patients with cirrhosis had more severe form of COVID-19, lower blood oxygenation and needed treatment by O_2_-therapy (p<0.046). Death rate was 12%. A strong correlation was found between the need for O_2_-therapy and deaths (p<0.001) and between characteristic findings for COVID-19 in pulmonary CT imaging and low blood oxygenation (p<0.003).

Conclusion: Comorbidity with chronic diseases, such as liver cirrhosis, has an important impact on the severity and mortality of the patients with COVID-19 infection. Inflammatory indices, such as NLR (neutrophil to lymphocyte ratio) and MLR (monocyte to lymphocyte ratio), are useful tools in predicting the evolution toward severe forms of the disease.

## Introduction

The pandemic caused by the novel severe acute respiratory syndrome coronavirus 2 (SARS-CoV-2) virus has led to the disease termed COVID-19 by the WHO (World Health Organisation) [1]. Studies indicate that it must be considered a systemic disease because it can affect multiple organ systems, including the gastrointestinal system [2]. 

The pandemic found unprepared every care worker and the health system itself. It was also a challenging time for all the patients with chronic diseases, who needed periodic and long term follow up. During this period, there was a lot of emerging information about this novel virus, much of which is important and clinically relevant to gastroenterologists who daily face vulnerable and fragile patients suffering from chronic diseases.

On this day in Albania, more than 332000 cases of COVID-19 are reported and 3596 deaths are attributed to the disease [3]. The objective of this study is to observe the overall impact of the pandemics COVID-19 in patients with digestive diseases hospitalized at the Department of Gastroenterology-Hepatology in “Mother Teresa” University Hospital Center during the period June 2020 (when the first case of COVID-19 was diagnosed) to December 2021.

## Materials and methods

A retrospective study was carried out from June 2020 (when the first case of COVID-19 was diagnosed) to December 2021 in the Gastroenterology-Hepatology Department at “Mother Teresa” University Hospital Center (UHC) in Tirana. Two thousand five hundred and twenty-seven hospitalizations were registered during this time in the same Department, 180 cases were tested (according to the recommendations of the Albanian Ministry of Health), and 41 were confirmed positive for COVID-19 infection by Reverse Transcriptase-Polymerase Chain Reaction assays of nasopharyngeal swab specimens at Microbiological Laboratory in the same hospital.

In line with the recommendations of the Albanian Ministry of Health, the patients hospitalized for digestive diseases underwent RT-PCR test for every respiratory symptom or fever more than 37.5 degrees Celsius, with or without other symptoms. After testing positive and according to the severity of the disease, the patients were transferred to the Department of Infectious and Tropical Diseases in the same hospital for further and specialised treatment. They were transferred to other nurse assisted healthcare institutions or at home for auto-isolation if they had a mild form of the infection and didn't need specialized care. We collected the information from their medical record after they were discharged from our Department and transferred to the Department of Infectious and Tropical Diseases. The mean value of the hospital stay was calculated based on both hospitalisations (in the Department of Gastroenterology-Hepatology and in the Department of Infectious and Tropical Diseases).

Patients over 18 years of age admitted to the Department of Gastroenterology-Hepatology who tested positive for COVID-19 infection were enrolled in this study. We referred to the blood test performed after the patients resulted positive for COVID-19 infection. The questionnaire used included demographic data, urban or rural origin, blood group, BMI, drinking habit, the diagnosis of admission, vaccination for COVID-19, comorbidities, clinical signs, biochemical tests, length of hospital stay (in Gastroenterology-Hepatology Department and in the Department of Infectious and Tropical Diseases), transfer in specialized COVID-19 departments, other health care institutions or home. 

Severity of infection by COVID-19 was evaluated based on blood oxygenation/need for oxygen therapy and radiological data on pulmonary CT imaging. The following systemic inflammation indices were calculated in all patients: NLR (neutrophil to lymphocyte ratio = absolute neutrophil count/absolute lymphocyte count); d-NLR (derived neutrophil to lymphocyte ratio = absolute neutrophil count/white blood cells-absolute neutrophil count); MLR (monocyte to lymphocyte ratio = absolute monocyte count/absolute lymphocyte count); PLR (platelet to lymphocyte ratio = absolute platelet count/absolute lymphocyte count); SII (systemic immune-inflammation index = absolute platelets count x NLR); SIRI (systemic inflammation response index = absolute neutrophil count x absolute monocyte count/absolute lymphocyte count). 

All data collected from the patient’s records were analyzed by IBM SPSS Statistic version 25 (Statistical Package for Social Sciences, version 25; IBM Corp., Armonk, NY). Frequency distributions (absolute numbers and corresponding percentages) were presented for all categorical variables. Mean value and standard deviation were calculated for all numerical variables. Student t-test (independent samples t-test) was used to compare the average values ​​of continuous variables between the two groups. For the comparison of categorical variables, the Chi-square test of association was used. In all cases, p≤0.05 values ​​were considered statistically significant.

## Results

The patients who were positive for COVID-19 infection made 1.6% (41 patients) of the total hospitalized cases (2527) in the Department of Gastroenterology-Hepatology in “Mother Teresa” UHC, during the period of our study. The mean age of the patients was 60.05 +/- 15.008 years. The predominant group of age (48.8%) was between 41-60 years old. The number of infected men was significantly higher than women (p<0.001). There was no difference between the blood groups of the infected patients (Table 1). Twenty-one percent of the patients were vaccinated at the time of the diagnosis. Most of the patients came from urban areas and more than a half came from the capital, Tirana.

**Table 1:** Patient’s characteristics

	NUMBER OF PATIENTS	PERCENTAGE
GENDER		
Female	13	31.7
Male	28	68.3
AGE GROUP		
<20	1	2.4
21-40	2	4.9
41-60	20	48.8
>61	18	43.9
BLOOD GROUP		
0	13	31.7
A	12	29.2
B	6	14.6
AB	0	0.0
COMORBIDITIES		
Yes	30	73.2
No	11	26.8
VACCINATED	9	21.9

The frequency of the digestive diseases in the infected patients was: cirrhosis 31.7%, acute pancreatitis 21.9%, alcoholic liver disease 21.9%, gastrointestinal hemorrhage 19.5%, digestive cancer 14.6%, biliary diseases 7.3%, inflammatory bowel disease 2.4%, other digestive diseases (one case of irritable bowl syndrome with diarrhoea, one case of diverticulitis) 4.8%. Fever (90%) and fatigue (78.04%) were the most dominant clinical signs at the diagnosis of COVID-19 infection (Table 2). 

**Table 2:** Clinical signs at the diagnosis of COVID-19 infection

CLINICAL SIGNS	FREQUENCY	PERCENTAGE
Fever	37	90.00
Fatigue	32	78.04
Abdominal pain	20	48.78
Cough	17	41.46
Headache	14	34.14
Dyspnea	8	19.51
Vomiting	8	19.51
Sore Throat	6	14.63
Anosmia	1	2.43
Diarrhea	0	0.00

Some patients hospitalized for digestive diseases had one or more comorbidities. They were as follows: hypertension 43.9%, anemia 15%, diabetes 14.63%, renal diseases 14.63%, other cardiovascular diseases (except hypertension) 12.19%, neurological diseases 12.19%, pulmonary diseases 7.31% (Tables 3-4). 

**Table 3:** Comorbidities in patients hospitalized for digestive diseases

COMORBIDITIES	FREQUENCY	PERCENTAGE
Hypertension	18	43.9
Anemia	15	36.58
Diabetes	6	14.63
Renal disease	6	14.63
Cardiovascular diseases (other than hypertension)	5	12.19
Neurological diseases	5	12.19
Pulmonary diseases	3	7.31

**Table 4:** Number of comorbidities per patient in patients hospitalized for digestive diseases

COMORBIDITIES/ PATIENT	FREQUENCY	PERCENTAGE
0	11	26.8
1	13	31.7
>2	17	41.5

The mean hospital stay was 11.9 days ± 8.558 SD. The distribution in time of the infected cases in the patients hospitalized in our Department was similar to those in the non-hospitalized patients, according to the data referred by the Albanian Institute of Public Health [3]. 

The study of the biochemical and hematological parameters showed elevation of the average value of AST, ALT (AST value higher than ALT, p<0.001), and bilirubin in all patients. Their comparison in the patients who survived and those who didn’t show higher levels of creatinine in the fatality cases (Table 5).

**Table 5:** Differences in hematological and biochemical parameters in deceased and recovered patients RBC: Red blood cells; MCV: Mean corpuscular volume; WBC: White blood cells; AST: Aspartate aminotransferase; ALT: Alanine transaminase; ALP: Alkaline phosphatase; CRP: C-reactive protein

VARIABLE	TOTAL		RECOVERED		DECEASED		p VALUE
	MEAN	SD	MEAN	SD	MEAN	SD	
RBC	3650975.61	883317.05	3667777.78	855663.8	3530000.0	1172710.5	0.748
Hematocrite	32.48	7.30	32.69	7.25	31.0	8.28	0.633
MCV	89.84	11.99	89.63	9.97	91.32	23.76	0.773
WBC	9063.41	4631.34	8886.11	4735.83	10340.00	3990.90	0.518
Neutrofile	7039.02	4060.5	6794.44	4057.01	8800.0	4055.2	0.307
Lymphocytes	1321.95	959.56	1391.67	1000.39	820.0	303.31	0.216
Monocytes	607.32	452.43	602.78	455.12	640.0	482.70	0.886
Eosinofiles	163.64	281.53	182.14	300.68	60.00	89.44	0.380
Basofiles	15.63	36.89	17.86	39.002	0.0	0.0	0.374
Trombocytes	198853.66	110268.43	203138.89	109935.08	168000	120357.8	0.511
AST	89.46	90.56	89.08	95.47	92.20	47.28	0.944
ALT	85.80	104.05	90.39	109.28	52.80	49.18	0.456
ALP	160.52	164.39	148.00	145.80	226.25	260.02	0.394
Bilirubine	1.9135	3.44	1.7986	3.63	2.7180	1.510	0.583
Glicemia	136.82	75.022	136.06	78.62	142.0	49.34	0.871
Urea	50.72	50.38	45.26	44.57	90.06	76.14	0.062
Creatinine	1.3280	1.44	1.0650	0.78	3.22	3.20	0.01
Amylase	212.33	353.56	239.06	376.48	52.00	17.321	0.410
Lipase	329.94	621.95	388.54	680.24	76.00	76.41	0.452
CRP	8.77	8.63	9.24	9.077	5.31	2.87	0.471
D-Dimer	258.78	751.63	313.23	822.94	4.72	4.17	0.536
ProtrombinT	67.12	24.47	68.46	25.31	56.33	14.97	0.429
Albumin	3.83	5.06	4.04	5.34	2.13	0.25	0.548

**Figure 1:**
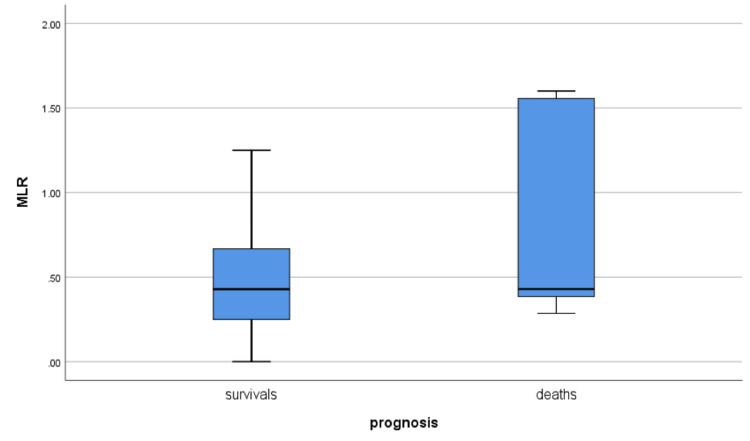
MLR correlation to prognosis in patients with digestive diseases and COVID-19 infection MLR: Monocyte to lymphocyte ratio; COVID-19: Coronavirus disease 2019

**Figure 2:**
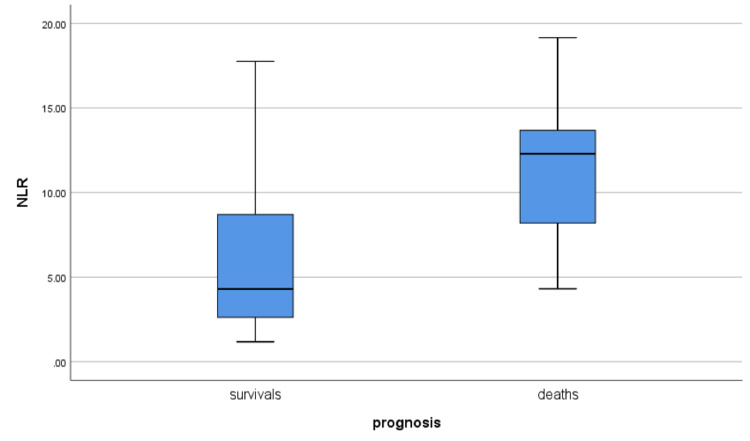
NLR correlation to prognosis in patients with digestive diseases and COVID-19 infection NLR: Neutrophil to lymphocyte ratio; COVID-19: Coronavirus disease 2019

Lymphopenia was present in 41.46 % of all patients, while it was found in 80% of the deceased. After the analysis of six systemic inflammation indices, we observed a significant predictive value of NLR and MLR indices for the non-survivals (Table 6, Figures 1-2).

**Table 6:** Inflammatory markers and correlation to non-survivals NLR: Neutrophil to lymphocyte ratio; d-NLR: Derived neutrophil to lymphocyte ratio; MLR: Monocyte to lymphocyte ratio; PLR: Platelet to lymphocyte ratio; SII: Systemic immune-inflammation index; SIRI: Systemic inflammation response index

INFLAMMATORY MARKERS	TOTAL	RECOVERED	DECEASED	p-VALUE
NLR	6.6307+/-4.75227	5.9515+/-4.27905	11.5206+/-5.62010	0.012
d-NLR	0.8972+/-0.06258	0.8957+/-0.06147	0.9080+/-0.07704	0.685
MLR	0.5201+/-0.36728	0.4742+/-0.29276	0.8509+/-0.66575	0.030
PLR	185.6607+/-121.50578	180.1320+/-113.36343	225.4672+/-181.47222	0.441
SII	1312048.4557+/- 1085603.31717	1193352.3366+/-907751.68993	2166660.5128 +/-1880287.10621	0.059
SIRI	4214.4188+/- 4507.97782	3798.4442 +/-4031.65844	7209.4359 +/-6944.33631	0.114

One of the most important findings in our study was that the patients with hepatic cirrhosis were the group with more severe form of COVID-19, who had lower blood oxygenation values and who needed treatment by oxygen therapy (p<0.046) (Table 7). Acute pancreatitis, even in his severe form according to Balthazar classification, was not associated with severe form of infection by COVID-19.

**Table 7:** Diagnosis at the hospitalization and need for treatment by oxygen therapy IBD: Inflammatory bowel disease

DIAGNOSIS	NO O_2_-THERAPY	O_2_-THERAPY	p-VALUE
Cirrhosis	9	4	0.046
Pancreatitis	9	0	0.160
Gastrointestinal hemorrhage	7	1	0.849
IBD	1	0	0.675
Gastrointestinal cancer	4	1	0.717
Biliary disease	3	0	0.456
Hepatocarcinoma	1	0	0.675
Other digestive diseases	2	0	0.548

About 43.90% of the patients had typical radiological signs of COVID-19 in the pulmonary CT scan. Death rate in our study was 12% (five patients), and 80% of the deceased patients were men. A strong correlation was found between the need for oxygen therapy treatment and deaths (p<0.001) and between characteristic findings for COVID-19 in pulmonary CT imaging and low blood oxygenation (p<0.003). Deaths were not higher in the non vaccinated group. Significant correlation wasn’t found between the presence of comorbidities, older age of the patients, and deaths. 

After the positive diagnosis of COVID-19 infection, 19.51% of the patients (with moderate and severe clinical manifestations) were transferred to the Department of Infectious Diseases in “Mother Teresa” UHC, while the other cases were transferred to other general healthcare institutions or to home for auto-isolation.

## Discussion

Our study has limitations, mostly related to his retrospective design and the small group of patients, which probably doesn’t reflect the real situation of COVID-19 infection in the patients affected by gastrointestinal diseases. 

Gastrointestinal and hepatic involvement during COVID-19 infection have been recognized and are mediated by the expression of angiotensin-converting enzyme 2 (ACE2) on the gastrointestinal tract, the main receptor of SARS-CoV-2 [4-5], so we tried to describe the effects of this newly emerging infection in already ill patients. During the first year of the pandemic in our country, access to the PCR testing, even for the hospitalized patients, was not for everyone, which might explain the low percentage of positive patients in the study (1.6%). 

The group of age mostly affected, 41-60 years old, is concordant to other studies [6]. Men are more affected than women in our study, and they have higher death rates. According to similar studies, men might also have more severe forms of disease and the higher number of deaths is explained by the fact that testosterone is known to suppress the immune system, while estrogen can promote it; this could be why women have a stronger immune response against bacteria and viruses [7-9].

The inner migration of the last 30 years in Albania, from the rural to the urban areas and from the small cities to towards the capital, explains the highest number of cases coming from the urban areas and more than half of them from Tirana. “Mother Teresa” UHC is also the only tertiary center and the Department of Gastroenterology-Hepatology is unique in Albania. This unit hosts most of the gastrointestinal cases from all over the country. Cirrhosis is the pathology with the highest number of hospitalizations in our Department and unsurprisingly, the patients with cirrhosis made most of the cases in the infected group. 

Hyperthermia (>37.5 degrees Celsius) was the most frequent clinical sign found in patients who tested positive for COVID-19, and not without reason. At the beginning of the pandemic, the guidelines of the Albanian Ministry of Health about “whom to test” for the hospitalized patients suggested testing patients with respiratory clinical symptoms and every febrile patient.

The presence of one or more non-digestive comorbidities (Table 3) in our group of patients supports the fact that patients with digestive diseases, mostly the chronic ones, are complex, difficult to treat, and with high risk of fatalities, not only when faced to COVID-19 infection. Surprisingly in our study we didn’t find any statistically important correlation between the presence of non-digestive comorbidities, older age, and the non-survivors. Even though now it is well established that age and presence of comorbidities are important risk factors for moderate and severe forms of the infection [10-11].

Meanwhile, in our group of patients, cirrhosis was found related to severe form of disease caused by COVID-19 and death, which is concordant with other studies that show increased risk of decompensation and mortality in those patients [6,12].

It is established that hepatocytes constitute an important source of proteins involved in innate and adaptive immune responses. So, liver injury can lead to compromised immune surveillance by reducing the hepatic synthesis of these proteins. It is well known that chronic liver disease (CLD) and cirrhosis are characterized by immune dysregulation. In patients with decompensated cirrhosis, cirrhosis-associated immune dysfunction can switch from predominantly pro-inflammatory to predominantly immunodeficient, leading to increased susceptibility to infections and it is unsurprising that patients with CLD, especially those with decompensated cirrhosis, are at higher risk of COVID-19-related morbidity and mortality [13-14].

The analysis of hematological and biochemical parameters in our group showed elevation of hepatic enzymes and bilirubin, regardless the diagnosis. Referring to two systematic reviews with meta-analysis [15-16], liver function tests (aspartate and alanine aminotransferases and bilirubin) were abnormal in 15-19% of patients with COVID-19. Available evidence suggests that, in this subset of patients, liver injury can result from direct pathogenic effects by the virus or systemic inflammation with a complicated disease course [17].

During the COVID-19 pandemic many indices and scores of systemic inflammation are developed and used, mostly in intensive care units, to predict the severe forms of disease and mortality. In our study we used some of these indices and found a correlation between cellular phenotypes of the white series, used in the indices of systemic inflammation as NLR (neutrophil to lymphocyte ratio) and MLR (monocyte to lymphocyte ratio), which were predictive of severe COVID-19 and strongly related to the mortality (respectively p<0.01, p<0.03). According to multiple studies, NLR has a prognostic value in many systemic diseases because it is strongly related to systemic inflammatory status [18-20]. Recently NLR has also been included in a risk score for the evaluation of the gravity in the patients with COVID-19 [21-25]. The value of this indice is supported by the fact that neutrophils are the first immune cells activated during antiviral immune responses and they represent 50-70% of all circulating leucocytes [26].

Lymphopenia was also found in most of the deceased patients in our study. Referring to different studies, in cases of severe COVID-19, lymphopenia is a common feature related to immune hyperactivity, and it suggests a state of systemic self-destructive inflammation, in which, although the T cell count decreases, T cells exhibit a hyperactivated phenotype [27-28]. The hematological indices we used are low cost markers and accessible to all patients, so they must be used to predict and treat the severe forms of COVID-19 infection correctly. 

## Conclusions

Regardless of several limitations of this study, such as the small number of patients and its retrospective design, it brings for the first time data concerning patients with gastrointestinal diseases and COVID-19 infection hospitalized in the only tertiary center in Albania.

The study highlights the fact that the comorbidity with chronic diseases, such as liver cirrhosis, has an important impact on the severity and mortality of patients with COVID-19 infection. Inflammatory indices, such as NLR (neutrophil to lymphocyte ratio) and MLR (monocyte to lymphocyte ratio), recently developed to assess the systemic inflammatory status, are useful tools in predicting the evolution toward severe forms of the disease.
